# Myelogenic Leukaemia

**Published:** 1915-02

**Authors:** 


**Myelogenic Leukaemia** treated with benzol and X-rays. Harry B. Smith of Ann Arbor, "Jour. Mich. State Med. Soc.," January, 1915. Male 32, Nov. 23, 1913, reds 3,310,000; 492,000; Hb. 44%; 60% of whites myelocytes. Benzol, ½ G. per diem, increased to 5 G. by Dec. 19, 1913. Jan. 15-Sept. 1, 1914, daily dose 12.5 G. Leucocytes had gradually diminished to 23,000 by May 5, Hb. 80%; myelocytes 28% Apr. 7. When seen Sept. 1, 1914, whites had increased to 373,000 when benzol was discontinued and X-Rays begun. Nov. 5, 1914, reds 4,500,000; whites 70,000; Hb. 65%. Author advises separate use of benzol and X-Rays, according to results.

